# Pilot: Wnt signaling controls *pgam2* expression in zebrafish cardiomyocytes

**DOI:** 10.17912/micropub.biology.002294

**Published:** 2026-08-10

**Authors:** Madisyn A. Hildenbrand, Klejdi Lako, Ugo Coppola

**Affiliations:** 1 Department of Biological Sciences, Florida Gulf Coast University, Fort Myers, FL, United States; 2 Department of Biological Sciences, University of South Carolina, Columbia, SC, United States

## Abstract

The relationship between developmental signaling pathways and basal metabolism in zebrafish hearts is still unexplored. Given the key role of canonical Wnt signaling in zebrafish cardiac development, our study analyzes the responsiveness to Wnt of *phosphoglycerate mutase 2* ( *pgam2* ), which catalyzes the glycolytic conversion of 3-phosphoglycerate to 2-phosphoglycerate. Notably, Pgam2 emerged as a gateway between glycolytic and cholesterol metabolism for its intersection with ATP generation within the cell. Since Pgam2 is linked to overall metabolic homeostasis and multiple cardiovascular diseases (CVDs), it represents a perfect candidate to dissect the Wnt involvement in the basic metabolism of developing cardiomyocytes.

**Figure 1. Wnt pathway regulates pgam2 gene expression in zebrafish cardiomyocytes:** **A)** Differential expression of *pgam2* gene in WT whole embryos vs WT isolated hearts at 48 hpf. **B)** Effects of BIO/XAV939 treatments on *pgam2* expression in WT whole embryos at 48 hpf. **C)** Effects of BIO/XAV939 treatments on *pgam2* expression in isolated hearts at 48 hpf. **D)** Combined effect of BIO and DEAB treatment on *pgam2* expression in WT whole embryos at 48 hpf. **E)** Combined effect of BIO and DEAB treatment on *pgam2* expression in isolated hearts at 48 hpf. Fold difference is relative to *β-actin* . Error bars in the graph indicate s.e.m. * indicates P < 0.05, ** indicates P < 0.01, **** indicates P < 0.0001. **F)** Schematic showing the relationship between Wnt and *pgam2* , with a limited contribution from retinoic acid (RA).

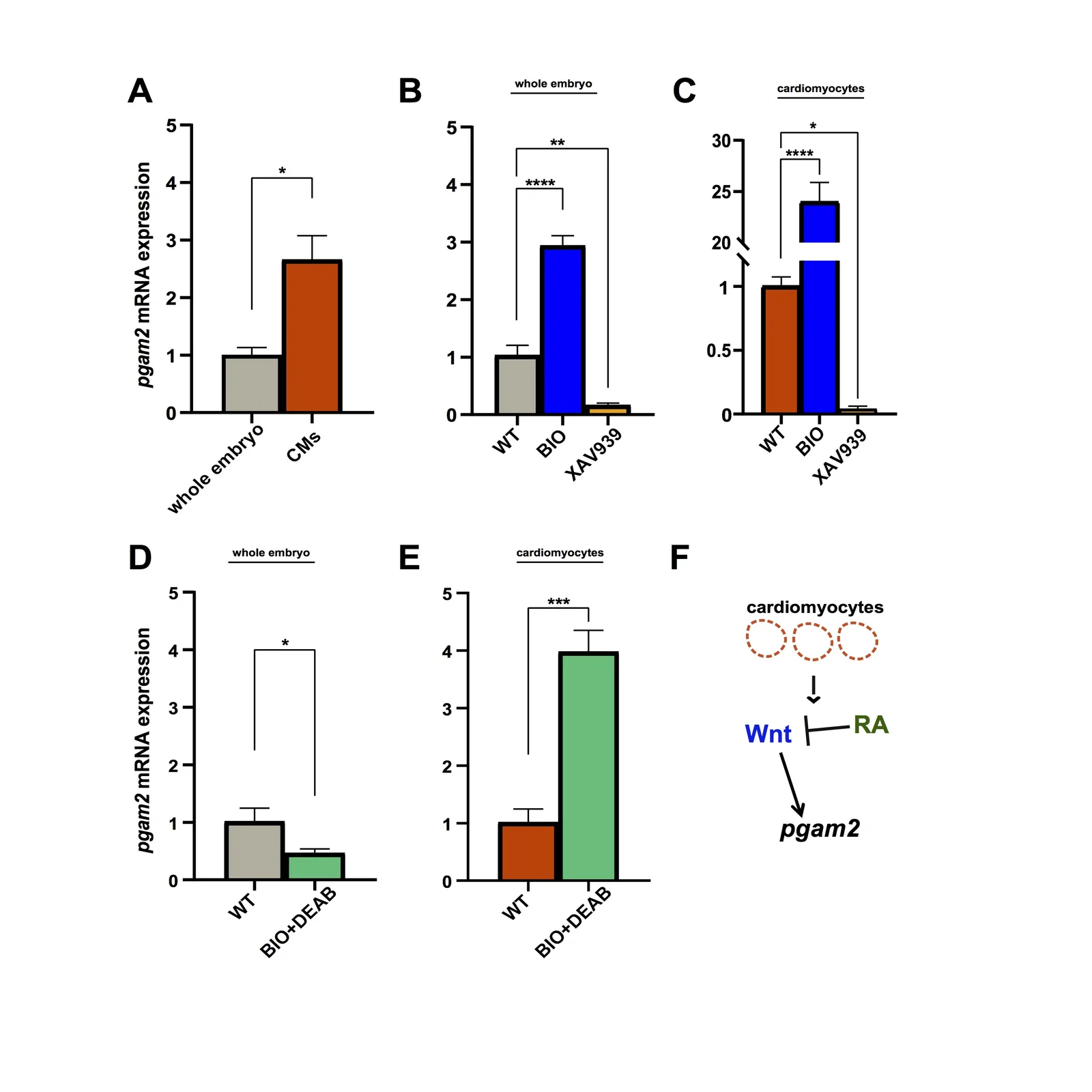



## Description

Zebrafish emerged as a model for the investigation of cellular metabolism, with multiple direct implications in the context of metabolic diseases (Benchoula et al. 2019) . While recently connected (Iwata and Vanderhaeghen 2024) , the relationships existing between developmental pathways and metabolic processes are still very obscure. Intriguingly, Wnt pathway disruptions have been associated with multiple metabolic defects in mammals (Abou Azar and Lim 2021) , while mutations in the transcription factor *TCF7L2* cause severe metabolic phenotypes in mice (Nguyen-Tu et al. 2021) . With respect to the metabolic aspects in cardiac development and disease, zebrafish has been indicated as a key model for its versatility (Angom and Nakka 2024) . Additionally, canonical Wnt signaling represents a fundamental asset in the regulation of zebrafish cardiac development (Ueno et al. 2007) , with a conserved role in mammalian models (Liang et al. 2020) . Therefore, in light of its key role in the regulation of critical steps in cellular metabolism (Guo et al. 2024) , we tested the impact of Wnt signaling on *pgam2* (Singh et al. 2023) through an established pharmacological approach (Coppola et al. 2024) in both WT whole-embryos and cardiomyocytes (Figure 1). First, we compared the expression level of *pgam2* between whole embryos and isolated hearts from WT samples at 48 hpf ( Fig. 1A ), detecting a moderate *pgam2* increase in cardiomyocytes (CMs). To understand the impact of Wnt signaling on *pgam2* during early development, we measured its expression levels in embryos treated with both a Wnt activator (6-bromoindirubin-3'-oxime, BIO) and a Wnt inhibitor (3,5,7,8-tetrahydro-2-[4-(trifluoromethyl)phenyl]-4H-thiopyrano[4, 3-d]pyrimidin-4-one, XAV939) ( Fig. 1B ). *Pgam2* resulted in an increase in BIO-treated samples, while was reduced with the XAV939 treatment ( Fig. 1B ). In order to analyze this relationship in CMs, we performed the same treatments on *myl7: EGFP* transgenic embryos and measured *pgam2* expression levels in isolated hearts ( Fig. 1C ). Intriguingly, the effect of BIO on *pgam2* expression in CMs is very strong, with a net increase in comparison with WT whole embryos ( Fig. 1B ), suggesting a stronger effect of Wnt on this key metabolic gene in the heart. Since retinoic acid (RA) represents a significant pathway in the homeostasis of early vertebrate embryos and their hearts (D’Aniello et al. 2013) and a general regulator of metabolic balance (El Haddad et al. 2017) , we tested the RA effect on the Wnt- *pgam2* relationship through a functional epistatic approach ( Fig. 1D- E). Combining the effect of BIO with the RA inhibitor 4-diethylaminobenzaldehyde (DEAB) in WT whole embryos caused a reduction in *pgam2* expression ( Fig. 1D ). On the other hand, the same epistatic approach caused a decrease of *pgam2* expression ( Fig. 1E ) when compared to BIO alone in CMs ( Fig. 1C ). Intriguingly, the effect of RA inhibition is similar between WT and isolated hearts, suggesting a synergic role of Wnt and RA pathways in the regulation of *pgam2* during zebrafish development. Here, we report the first study illustrating the relationship between the Wnt pathway and Pgam2 in zebrafish hearts ( Fig. 1F ). While the role of Wnt in the metabolic reprogramming in tumors has been already indicated (Koushyar et al. 2022; Tümen et al. 2024) , the relationship between Wnt and basic metabolism in zebrafish heart and cardiovascular pathologies has not been clarified. For its well-known versatility as a model system in development and disease (Westerfield 2007) and the recent gain of significance in metabolic studies (Benchoula et al. 2019; Abou Azar and Lim 2021) , zebrafish represent a valid target to study metabolic physiological interactions. Through well-established pharmacological manipulations (Coppola et al. 2024; DeWildt et al. 2026) , we highlighted a clear control exerted by Wnt on *pgam2* during zebrafish early development. Specifically, we registered higher *pgam2* expression in CMs and higher effect of Wnt on gene expression in isolated hearts, suggesting a more relevant effect/control in cardiovascular development. Intriguingly, our epistatic approaches unveiled a contribution of the RA pathway in the relationship existing between Wnt and *pgam2* in zebrafish CMs, suggesting a potential RA-Wnt interaction in the control of *pgam2* expression in the heart, with Wnt playing a major role in this context ( Fig. 1F ). Additionally, since Pgam2 has a significant role in the control of the balance between glycolytic pathway and cholesterol metabolism (Guo et al. 2024) , our findings pave the way to further experiments towards the understanding of Wnt impact of general metabolism in the heart. Furthermore, both the incidence of Wnt defects on mammalian metabolic homeostasis (Abou Azar and Lim 2021) and the implication of *TCF7L2* mutations in murine overt metabolic phenotypes (Nguyen-Tu et al. 2021) align with our pharmacological approach. Hence, our zebrafish data speak in favor of a conserved Wnt involvement in the regulation of a key metabolic gene, *Pgam2* , in the context of heart development. Intriguingly, human *PGAM2* gene has been associated with Glycogen Storage Disease Type X (GSD X) (Tsujino et al. 1993; Hadjigeorgiou et al. 1999; Nayab et al. 2021) , which severely affects muscle basic functionalities. Furthermore, BIO activates Wnt signaling through the inhibition of the Glycogen synthase kinase 3 (Gsk3) (Law and Zheng 2022) , which is a pivotal element of balance in the cellular metabolism (Wang et al. 2022) . On the other hand, XAV939 effectively blocks Wnt signaling interacting with Tankyrases (Wu et al. 2016) , which in cancer cells promote aerobic glycolysis (Yang et al. 2019) . Thus, it is tempting to speculate a control exerted by Wnt signaling on the basal metabolism in the heart through Pgam2. In cancer cells, both Wnt signaling (Flores-Hernández et al. 2025) and PGAM2 (Gizak et al. 2015; Wang et al. 2025) impact basal metabolism, highlighting a molecular and functional relationship existing in multiple tissues. Furthermore, the *Pgam2* overexpression causes cardiac weakness in mice (Okuda et al. 2013) and *PGAM2* resulted in overexpression in patients affected by heart failure (Li et al. 2021) , both supporting its involvement in the regulation of basic cardiac development. With respect to *PGAM2* implication in cardiac defects, it has been identified as a marker for cardiac ischemia (Li et al. 2012) and associated with cardiac hypertrophy (Li et al. 2025) . Additionally, *N* ^6^ -methyladenosine (m6A) post-transcriptional modifications cause glycolytic impairments in the heart of murine obesity models (Zhang et al. 2021) . Henceforth, in light of the significance of *Pgam2* orthologs in cardiac development and disease and for its strategic position among distinct metabolic pathways (Guo et al. 2024) , future studies will aim to completely decipher the role of Wnt in regulating *pgam2* and other genes within specific metabolic pathways (glycolysis, cholesterol, TCA), shedding light on novel Wnt-dependent gene regulatory networks (GRNs) in zebrafish (and vertebrate) hearts.

## Methods

*Zebrafish maintenance:* All zebrafish husbandry and experiments were performed following protocols approved by the Institutional Animal Care and Use Committee (IACUC) of Florida Gulf Coast University (# 2412000066A002). Adult zebrafish were raised and maintained following standard laboratory conditions and guidelines (Westerfield 2007) . All the WT fish used were mixed AB-TL backgrounds. The only transgenic line employed was *Tg(-5.1myl7: EGFP,* twu34Tg) (Huang et al. 2003) , whose ZFIN identifier is ZDB-TGCONSTRCT-070117-164. Embryos were raised at 28.5 °C in blue water and staged by hpf.

*Pharmacological approach:* Each drug treatment was carried out on embryos (20s-48 hpf stages) in 2 mL of blue water with drugs at specific concentrations in 3 mL glass vials (30 embryos/vial). Control and treated embryos were placed in vials on a nutator positioned in an incubator at 28.5˚C for the duration of the treatments. Drug concentrations were previously indicated (Coppola et al. 2024) : 2.5 μM DEAB (Sigma-Aldrich, Cat # D86256), 5 μM BIO (Tocris, Cat # 3194), 10 μM XAV939 (Tocris, Cat # 3748). All the drugs were dissolved in Dimethylsulfoxide (DMSO) (Sigma-Aldrich, Cat # D8418). All the drugs were washed employing blue water 3 times at 48 hpf before heart isolation procedure. Each experiment was performed in triplicate.

*Heart isolation:* The hearts from untreated (WT) and treated zebrafish at 48 hpf from the *Tg(-5.1myl7: EGFP)* line were isolated as previously described (Burns and MacRae 2006; Coppola et al. 2024; DeWildt et al. 2026) . ~500 embryos of the aforementioned transgenic line were anesthetized with tricaine and transferred to a sterile 1.5 mL microcentrifuge tube. They were then washed on ice 4 times in cold embryo disruption media (EDM, Gibco Leibovitz’s L-15 Medium, Cat # 11415114) with 10% fetal bovine serum (FBS, Gibco, Cat # 26140079). Embryos were placed in 1.25 mL EDM and triturated for approximately 1 minute using a syringe (1 sec rate) positioned on a ring stand. The fragments were transferred to a 105 μm mesh with 0.5 mL EDM, and immediately to a 40 μm mesh with additional EDM. Finally, the GFP+ hearts were selected utilizing an Olympus SZ51 Stereoscope and placed in clean EDM. The hearts were centrifugated and immediately pelleted at 1300 rpm for 5 minutes. RNA extraction was carried out employing the Single Cell RNA Purification Kit (Norgen Biotek, Cat # 51800). cDNAs were synthetised using iScript™ cDNA Synthesis Kit (Bio-RAD, cat #1708890). RNA was extracted using the same process on three different batches of 120 hearts.

*qPCR analyses:* experiments were performed as previously described (D’Aniello et al. 2013; Coppola et al. 2024; DeWildt et al. 2026) , employing Power SYBR Green PCR Master Mix (Applied Biosystems, Cat # 4368706) in a BioRad CFX-96 PCR machine. Expression levels were normalized against *β-actin* expression (D’Aniello et al. 2013) and results were analyzed using the Livak 2-ΔΔCT Method (D’Aniello et al. 2013) . Each experiment was performed in triplicate. *Pgam2* primers were: *pgam2fw* (AAAACCGTTTCTGCGGCTGG) and *pgam2rev* (CCATGTTTGGCTGCTGTCTC). The primer efficiency was 103 %. The significance of qPCRs was assessed with an ordinary one-way ANOVA with Dunnett’s correction for experiments with 3 samples (WT vs experimental condition) and Student’s t-test with Welch’s correction with 2 samples. p value <0.05 was considered statistically significant.
